# Plasma sphingolipids in HIV-associated chronic obstructive pulmonary disease

**DOI:** 10.1136/bmjresp-2017-000180

**Published:** 2017-04-03

**Authors:** Shane Hodgson, Timothy J Griffin, Cavan Reilly, Stephen Harvey, Bruce A Witthuhn, Brian J Sandri, Ken M Kunisaki, Chris H Wendt

**Affiliations:** 1 Department of Medicine, University of Minnesota, Minneapolis, Minnesota, USA; 2 Department of Biochemistry, Molecular Biology and Biophysics, University of Minnesota, Minneapolis, Minnesota, USA; 3 Division of Biostatistics, School of Public Health, University of Minnesota, Minneapolis, Minnesota, USA; 4 Department of Medicine, VAMC, Minneapolis, Minnesota, USA

**Keywords:** COPD ÀÜ Mechanisms, Immunodeficiency, COPD Pathology

## Abstract

**Introduction:**

Chronic obstructive pulmonary disease (COPD) is a significant cause of morbidity in persons living with HIV (PLWH) and HIV appears to uniquely cause COPD, independent of smoking. The mechanisms by which HIV leads to COPD are not clear. The objective of this study was to identify metabolomic biomarkers and potential mechanistic pathways of HIV-associated COPD (HIV-COPD).

**Methods:**

We performed case–control metabolite profiling via mass spectrometry in plasma from 38 individuals with HIV-COPD (cases), comparing to matched controls with/without HIV and with/without COPD. Untargeted metabolites of interest were identified with liquid chromatography with mass spectrometry (LC-MS/mass spectrometry (MS)), and targeted metabolomics for tryptophan (Trp) and kynurenine (Kyn) were measured by selective reaction monitoring (SRM) with LC-MS/MS. We used mixed-effects models to compare metabolite concentrations in cases compared with controls while controlling for relevant biological variables.

**Results:**

We identified 1689 analytes associated with HIV-COPD at a false discovery rate (FDR) of 10%. In PLWH, we identified 263 analytes (10% FDR) between those with and without COPD. LC MS/MS identified Trp and 17 lipids, including sphingolipids and diacylglycerol. After adjusting for relevant covariates, the Kyn/Trp ratio measured by SRM was significantly higher in PLWH (p=0.022), but was not associated with COPD status (p=0.95).

**Conclusions:**

There is a unique metabolite profile in HIV-COPD that includes sphingolipids. Trp metabolism is increased in HIV, but does not appear to independently contribute to HIV-COPD.

**Trial registration numbers:**

NCT01810289, NCT01797367, NCT00608764.

**Key messages**

- HIV-associated chronic obstructive pulmonary disease (HIV-COPD) has a unique plasma metabolomic profile that includes sphingolipids and fatty acids.
- Tryptophan catabolism is increased in PLWH, but does not correlate with COPD status.
- Additional studies are needed to determine how such metabolic pathways contribute to HIV-COPD and whether therapeutic interventions to alter sphingolipid expression can reduce the risk of COPD in persons living with HIV.

## Introduction

Combination antiretroviral therapy (ART) has markedly improved the survival of individuals living with HIV-1. However, improved survival has led to a higher prevalence of several chronic illnesses including chronic obstructive pulmonary disease (COPD), which affects an estimated 3–23% of persons living with HIV (PLWH).^1–8^ Although the prevalence of smoking is high in PLWH, observational studies suggest that HIV infection is an independent risk factor for accelerated lung function decline and subsequent COPD.^1^ ^2^ ^5^ In the era prior to the common use of highly active ART (HAART), pulmonary obstruction was mostly associated with advanced HIV/AIDS and frequent pulmonary infections.^9^ In the HAART era, COPD persists as a frequent comorbidity in PLWH even in the absence of AIDS or frequent pulmonary infections.^1–5^ ^8^ ^10–14^ It is highly likely that several factors are involved in HIV-associated COPD (HIV-COPD) and many investigators have implicated the HIV virus itself. Currently, the underlying mechanisms of HIV-COPD remain unclear and there is no biomarker to identify which patients with HIV will be at a higher risk for the subsequent development of COPD. For this study, we identified individuals in the Strategic Timing of Antiretroviral Treatment (START) study with HIV-COPD prior to the diagnosis of AIDS or initiation of HAART, offering a unique opportunity to characterise HIV-COPD.^15^

Recent developments in metabolomics and metabolic profiling have enabled the measurements of thousands of metabolites simultaneously in biological samples leading to new discoveries of biomarkers of disease.^16^ Metabolomics, the study of small molecules, is a field that complements genomics and proteomics to provide a snapshot into the physiology of human disease. As such, global, untargeted metabolomic profiling is a method for analysing complex diseases to understand the underlying pathways involved in disease pathogenesis and to identify biomarker candidates for disease. In this study, the primary aim was to perform large-scale untargeted analysis with mass spectrometry of plasma to identify metabolites associated with HIV-COPD. Our results revealed that HIV-COPD has a unique metabolomic signature and among the metabolome are sphingolipids.

## Methods

We performed a cross-sectional, matched case–control study using plasma samples from two large cohort studies.

### Study population

Cases were HIV positive and were selected from a pulmonary substudy of the START trial.^15^ ^17^ START enrolled HIV-positive, ART-naive persons with a CD4+ count >500 cells/mm^3^. From this cohort, we selected 38 participants with HIV-COPD (defined as forced expiratory volume in 1 s (FEV_1_)/forced vital capacity (FVC)< lower limits of normal and FEV_1_<80% predicted at baseline pulmonary function testing, table 1) for metabolomic profiling. HIV-positive controls consisted of 40 individuals with normal lung function (defined as FEV_1_/FVC>lower limits of normal and FEV_1_>80% predicted) matched on age, sex, region and smoking status. HIV-negative controls were identified in the Genetic Epidemiology of Chronic Obstructive Pulmonary Disease (COPDGene) study, an observational study that enrolled current or former smokers. COPDGene controls were matched on lung function, age, sex and race. These consisted of 17 individuals without COPD and 20 individuals with COPD (table 1). This study was approved by the University of Minnesota Institutional Review Board.

**Table 1:** Characteristics of cases and controls

	HIV(±) COPD(±) (n=38)	HIV(±) COPD(−) (n=40)	HIV(−) COPD(±) (n=20)	HIV(−) COPD(−) (n=17)
Age, years	38.97±9.21*	38.93±7.78*	48.18±3.67*	55.91±5.48
Sex	27M/11F	29M/11F	18M/2F	15M/2F
FEV_1_pp	70.95±11.6*	94.90±15.6*	73.08±12.8*	109.6±13.2
FEV_1_/FVC	0.663±0.0704*	0.815±0.0476	0.618±0.065*	0.818±0.0341
HIV RNA log10, copies/mL	3.95±1.04	3.86±0.994	NA	NA
CD4+ T-cell count, cells/mm^3^	707.03±198.89	716.26±182.84	NA	NA
CD8+ T-cell count, cells/mm^3^	1129.68±596.74	1142.86±912.3	NA	NA
CD4/CD8 ratio	0.793±0.395	0.936±0.722	NA	NA
Smoking status†	16/3/19	16/4/20	16/4/0	0/0/17
Race‡	15/22/1	16/22/2	15/5/0	15/2/0
Region§	6/13/17/2	8/13/17/2	20/0/0/0	17/0/0/0

*Significant from COPD(−) HIV(−) using Student's t-test.

†Smoking status (current/former/never).

‡Race (Caucasian/black/Latino).

§Region (North America/Europe/Africa/South America).

COPD, chronic obstructive pulmonary disease; FEV_1_pp, forced expiratory volume in 1 s, per cent predicted; FVC, forced vital capacity; NA, not available.

### Solvents and reagents

The acetonitrile (ACN) used in this study was of high pressure liquid chromatography (HPLC) grade and purchased from Fisher Scientific (Pittsburg, Pennsylvania, USA). HPLC grade methanol (MeOH) was acquired from Sigma-Aldrich (St Louis, Missouri, USA) and analytical grade formic acid from Fluka (Sigma, St Louis, Missouri, USA). The mass spectrometer lock-spray solution, leucine encephaline, was purchased from Waters (Milford, Massachusetts, USA). Ultra high purity bottled water was procured from Invitrogen (Grand Island, New York, USA).

### Sample preparation:

Aliquots of 100 µL of plasma had a heavy standard of 3 µL of 100 µM of kynurenine (Kyn)-D6 and 3 µL of 1 mM of tryptophan (Trp)-C11^13^ (Cambridge Isotope Laboratories, Tewksbury, Massachusetts, USA) added in prior to any preparation. The aliquots of plasma were then mixed with 400 µL of ice-cold solvent (100% MeOH), vortexed and placed on ice for 10 min. Samples were then centrifuged at 13 000×g for 10 min at 4°C, and the supernatant was removed and transferred into a clean low-retention phial. This step was repeated once. Samples were concentrated using a vacuum centrifuge to ∼50 µL. Formic acid was used to acidify the plasma samples which were added to the starting buffer used in ultraperformance liquid chromatography (5% ACN, 95% water, 0.1% formic acid) to ∼100 µL.

### MS analysis

For analyte profiling and quantitation, 10 µL of the undiluted sample was injected into Thermo Q-exactive LC-MS (ThermoFisher Scientific, Marietta, Ohio, USA) with Acquity ultra performance liquid chromatography (UPLC) C18 2.1×100 mm, 1.7 µm column (Waters, Milford, Massachusetts, USA) at 40˚C. All of the samples were subjected to a gradient going from buffer A to B over 15 min then flushing for 5 min. Buffer A consisted of 99.9% water with 0.1% formic acid, while buffer B was 99.9% ACN with 0.1% formic acid. To avoid batch effects, all samples were run in one continuous MS run. For quality control, internal standards consisted of blank samples spiked with four metabolites (phenylalanine-D6, hippuric acid-D5, Kyn-D6 and Trp-C11) and were placed at random intervals in the MS run. For quantitation, the individual samples were spiked with the same four metabolites for normalisation of the peak intensities. For metabolite identification, two samples from each group (±COPD, ±HIV) were run on the Thermo Q-exactive LC-MS in MS/MS data-independent acquisition mode with a 25 min of gradient A to B followed by 5 min of flushing.

### MS data processing

The .RAW files from the Q-Exactive were converted into mzXML using MSconvert (http://proteowizard.sourceforge.net/tools.shtml), and processed using the XCMS algorithm as implemented in the xcms package for R using the recommended pipeline (the symmetric family was used for retention time (RT) correction). Data from each sample were normalised to the mean of the heavy standards we introduced for Trp (mass/charge (m/z)=215.1231 and RT=1.841226) and Kyn (m/z=216.1318 and RT=1.941770), so that on the log scale the means of these two analytes were zero for all individuals.

### LC-MS/MS analysis for analyte identification

The .RAW files from Q-exactive LC-MS/MS were analysed using Thermo Xcalibur Qual Browser for fragmentation data of the analytes of interest. These analytes were matched to fragmentation spectra based on m/z and RT matching between LC-MS and LC-MS/MS. The fragmentation data for all of these analytes were compared and matched to metabolites via Metlin's MS/MS metabolite database within a 12 ppm difference.

### LC-MS/MS selective reaction monitoring analysis of Trp and Kyn

Diluted samples (20 µL, diluted 1:1000 for Trp and 1:100 for Kyn) were subjected to injection using an Agilent autosampler LC-MS/MS with an analytical Waters Symmetry C18, 3.5 µm column connected to the 5500 iontrap (Sciex, Framingham, Massachusetts, USA) fitted with a turbo V electrospray source. The samples were subjected to a linear gradient of 2% ACN, 0.1% formic acid to 98% ACN 0.1% formic acid for 10 min at a column flow rate of 250 µL/min. Transitions monitored are in online supplementary table 1S. The data were analysed using MultiQuant (Applied Biosystems, Foster City, California, USA) providing the peak area for the transitions. A standard curve was constructed using concentration ratios of Trp/Trp and Kyn /Kyn from fentomole to nanomole in 20 µL.

10.1136/bmjresp-2017-000180.supp1 supplementary table 1

### Statistics

To test for differences in metabolite concentrations, mixed-effects models were used that included fixed effects for HIV status, COPD status, the interaction between HIV and COPD status, FEV_1_ per cent predicted, race and smoking status and a random effect for match group. All matched groups were matched on age and sex. For analysis of individual metabolites, a logarithmic transformation was employed prior to model fitting, whereas the component of the targeted analysis which focused on the ratio of Kyn to Trp did not employ such a transformation. Otherwise, the same approach was employed for the analysis of the metabolites identified in the untargeted analysis as for the targeted analysis, except that we report an unadjusted p value for the targeted analysis, whereas we use the q-value approach to control the false discovery rate (FDR) at 10% for the untargeted analysis. V.3.2.3 of the statistical software package R was used for all statistical analyses.

### Analyte bioinformatics

MS/MS spectra .Raw files were observed using Thermo Xcalibur Qual Browser. MS/MS spectra for each analyte of interest were matched to metabolite spectra through the Metlin metabolite database from Scripps (https://metlin.scripps.edu/index.php). A degree of restraint, within 10% of the RT, was used when matching to feasible options based on RT of the analyte. Analytes illuted in the first 30s and after 15 min were eliminated.

## Results

### Study participant characteristics

Cases consisted of 38 individuals with HIV-COPD (table 1). HIV(+) controls were 40 individuals with HIV, but without COPD, matched on age, sex, smoking status, region and race. Baseline plasma samples were drawn prior to ART initiation or any AIDS-defining illness. All had CD4+ T-cell counts >500 cells/mm^3^ and there were no significant differences in plasma HIV RNA, CD4+ and CD8+ T-cell counts between HIV-positive cases and controls. The HIV(**−**) controls were significantly older than the HIV-positive participants.

### Metabolomics analyte profiles

**Figure 1:**
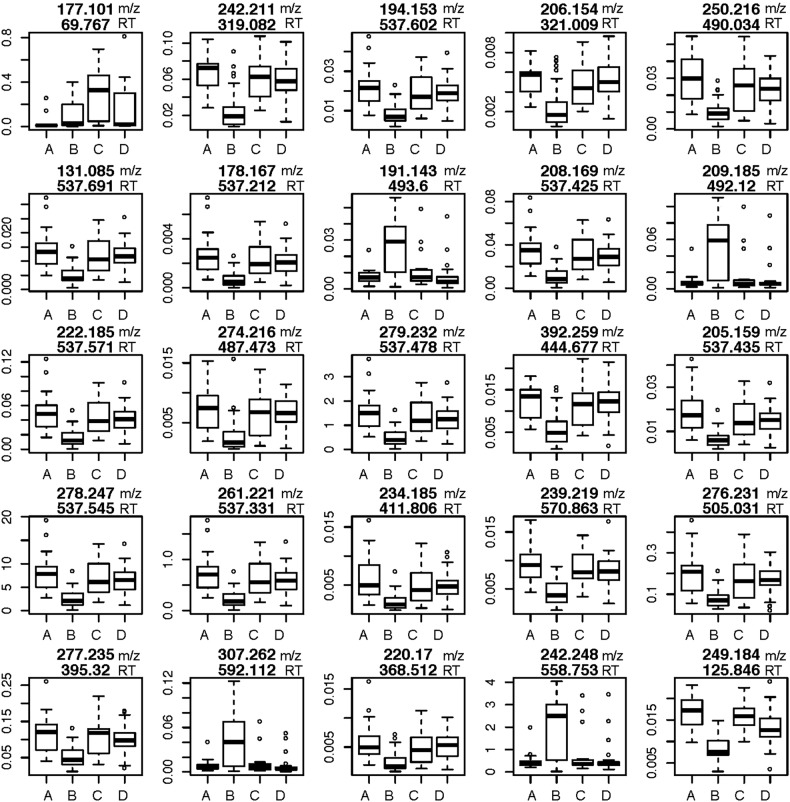
The top 25 analytes by relevant q-value differentially expressed in HIV with COPD. The top number is m/z and the bottom number is RT. Y-axis is intensity. (A) HIV− COPD−, (B) HIV+ COPD−, (C) HIV− COPD+, (D) HIV+ COPD+. COPD, chronic obstructive pulmonary disease; m/z, mass/charge; RT, retention time.

MS analysis detected 1689 plasma analyte signals, defined as a discreet m/z and RT, that correlated with a yet unknown metabolite. Since there is no universally accepted methodology to quantify analytes detected by mass spectrometry, we chose relative abundance as the sum of all peak intensities detected by the mass spectrometer that associated with the given analyte. Using a mixed-effects model with fixed effects for COPD status, HIV status, their interaction, FEV_1_ per cent predicted, race, sex and smoking status, we identified 880 analytes at 10% FDR that were attributable to the main effect of HIV status, that is, differences due to HIV averaging over COPD status; we identified no analytes that differed at 10% FDR for the main effect of COPD. Tests of the statistical interaction between HIV status and COPD status identified 263 analytes that displayed differences of interest (ie, a q-value <0.1). Of these 263 analytes, 232 (88%) also had significant main effects for HIV status. On examination of the estimated regression coefficients for HIV status and the interaction between HIV and COPD status of the 263 analytes, it was found that differences between HIV(+) and HIV(**−**) participants are, on average, smaller in COPD(+) participants compared with COPD(**−**) participants. This is reflected in figure 1 that displays the raw data for 25 analytes with the lowest q-values that met our FDR criterion for the interaction term. After eliminating isotopes and contaminants, 172 of the original 263 analytes differed based on HIV and COPD status and therefore qualified for further analysis described next.

### Metabolite identification

**Figure 2:**
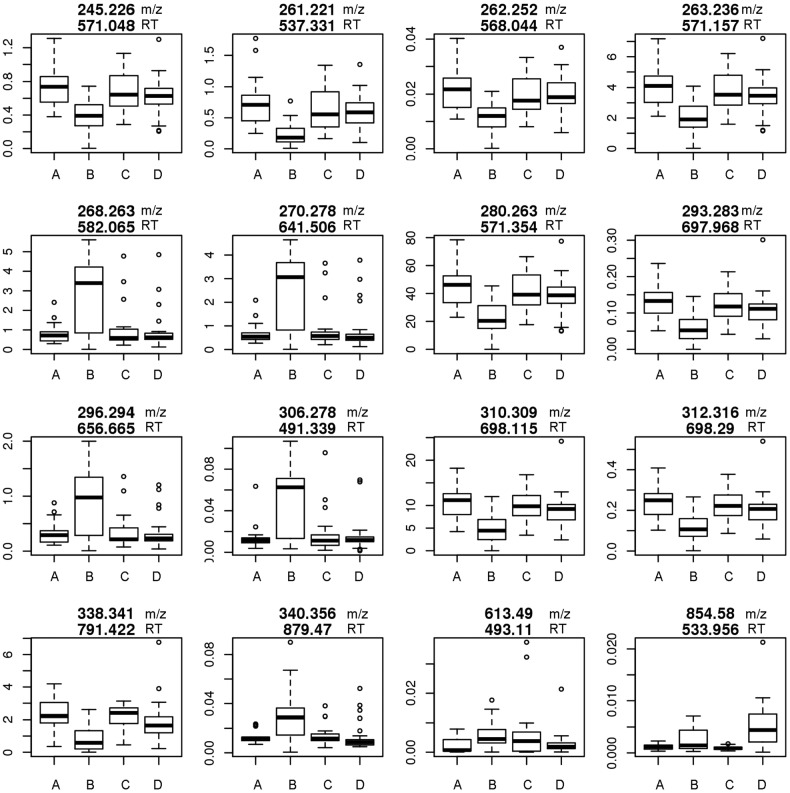
Lipids differentially expressed in HIV. The top number is m/z and the bottom number is RT. Y-axis is intensity. (A) HIV− COPD−, (B) HIV+ COPD−, (C) HIV− COPD+, (D) HIV+ COPD+. COPD, chronic obstructive pulmonary disease; m/z, mass/charge; RT, retention time.

To identify some of the significant analytes of interest, we performed LC-MS/MS targeting the 172 analytes we identified that differed in HIV-COPD. We were able to attribute 17 of these analyte spectra to lipids, including sphingolipids, diacylglycerol and fatty acids (table 2, figure 2, online supplementary table 2S). Ceramide (m/z 560.50948) concentrations were higher in HIV-COPD compared with +HIV/normal lung function, but did not quite meet our FDR threshold (q-value=0.11). Diacylglycerol was lower in HIV-COPD compared with +HIV/normal lung function and similar to those without HIV. We identified three lipids as fatty acids and one as a phospholipid. Generally, the fatty acids were higher in the HIV-COPD persons compared with +HIV/normal lung function, whereas the different sphingolipids demonstrated both increased and decreased concentrations.

**Table 2:** Metabolites identified by LC-MS/MS

m/z	RT (s)	Adduct	Accurate mass	Formula	Tentative identification	q-Value
245.22577	571.05	M+H-H_2_O	262.2297	C18H30O	Fatty acid	0.035141
261.22061	537.33	M+H-H_2_O	278.2246	C16H32O2	Fatty acid	0.002931
262.25198	568.04	M+H-H_2_O	279.2562	C18H33NO	Linoleamide	0.031476
263.23627	571.16	M+H	262.2297	C18H32O2	Fatty acid	0.023163
268.26277	582.06	M+H-H_2_O	268.2640	C17H35NO2	Sphingolipid	0.014423
270.27842	641.51	M+H-H_2_O	287.2824	C17H37NO2	Sphingolipid	0.010165
280.26257	571.35	M+H-H_2_O	297.2668	C18H33NO	Sphingolipid	0.046177
293.28267	697.97	M+Na	270.2923	C18H38O	Octadecanol	0.024386
296.29396	656.67	M+H-H_2_O	313.2981	C19H39NO2	Sphingolipid	0.021558
306.27791	491.34	M+Na	283.2875	C18H37NO	Stearamide	0.002926
310.30938	698.11	M+H-H_2_O	327.3137	C20H41NO2	Sphingolipid	0.021558
312.31609	698.29	M+H-H_2_O	329.3197	C20H43NO2	Sphingolipid	0.021623
338.34061	791.42	M+H	337.3345	C22H43NO	Docosenamide	0.005362
340.35618	879.47	M+H	339.3501	C22H45NO	Docosenamide	0.027448
560.50948	568.79	M+H-H_2_O	577.5070	C36H67NO4	Ceramide	0.113237
613.49004	493.11	M+H	612.4754	C39H65O5	Diacylglycerol	0.014779
854.57988	533.96	M+Na	831.5989	C45H86NO10P	Phospholipid	0.006038

m/z, Mass to charge ratio of metabolite; RT, retention time (s) in mass spectrometry column.

10.1136/bmjresp-2017-000180.supp2 supplementary table 2

### Targeted Trp and Kyn concentrations

**Figure 3:**
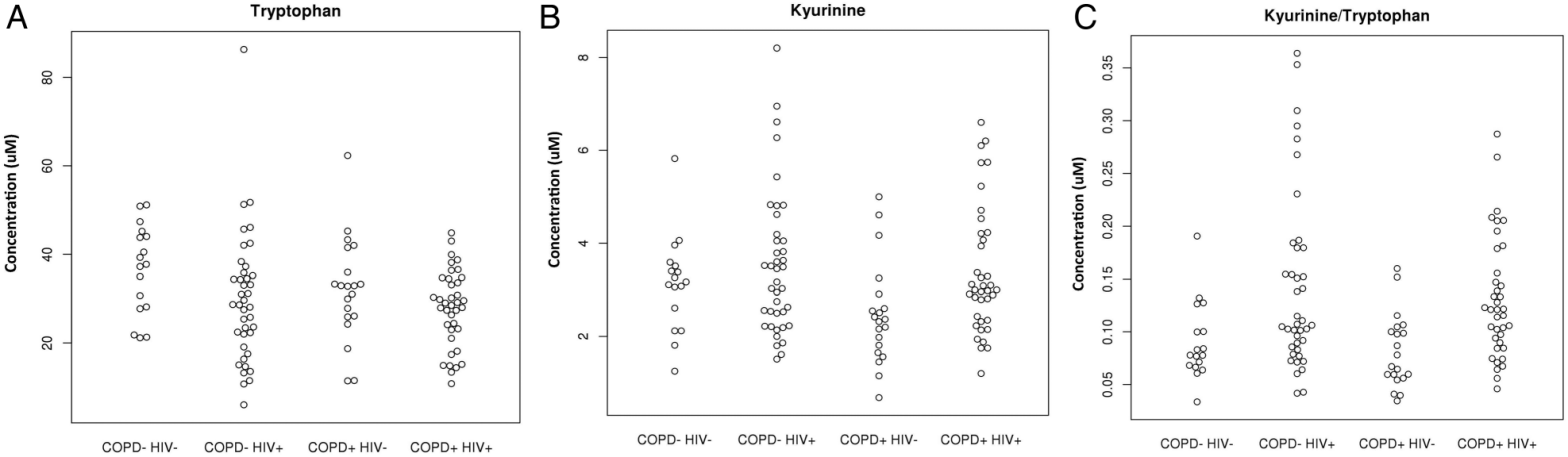
(A) Plasma tryptophan concentrations. (B) Plasma kynurenine concentrations. (C) IDO activity as reflected by kynurenine/tryptophan ratio. COPD, chronic obstructive pulmonary disease; IDO, indoleamine-2,3-dioxygenase.

One of the analytes differentially expressed in HIV+ participants was consistent with the essential amino acid Trp (m/z 205.097). Since Trp catabolism has been associated with both HIV infection and COPD,^18–20^ and standards are readily available, we sought to quantify Trp and its major metabolite, Kyn (m/z 209.094) by performing targeted selective reaction monitoring (SRM; figure 3). Indoleamine-2,3-dioxygenase (IDO) is the main inducible and rate-limiting enzyme involved in Trp catabolism, and IDO activity is expressed as the Kyn/Trp ratio. We found that Trp was lower in HIV(+) individuals compared with HIV(**−**) participants as previously reported,^21–23^ although this was not significant after adjustment for relevant covariates (figure 3A, p=0.0681). Trp was also lower in individuals with COPD, but this was not statistically significant (figure 3A, p=0.39). Kyn was higher in HIV-positive participants but was not statistically significant after adjustment for relevant covariates (figure 3B, p=0.5586), and was lower in COPD participants, albeit not significantly (figure 3B, p=0.1888). As a reflection of IDO activity, the Kyn/Trp ratio was significantly higher in HIV (figure 3C, p=0.022), but there was no difference due to COPD status (p=0.95).

## Discussion

A major gap in knowledge is the limited understanding of why PLWH are more susceptible to developing COPD independent of smoking status.^1^ ^2^ ^5^ ^10^ Currently, there is no biomarker to identify risk or lend insight into the mechanisms of developing rapid decline in lung function and subsequent COPD in PLWH. The pulmonary substudy of START offers a unique opportunity to identify biomarkers of HIV-COPD prior to the development of an AIDS-defining illness. This is important since pulmonary infection itself can lead to a loss of lung function.^24^ ^25^ In this exploratory study using an untargeted metabolomic approach, we identified 172 unique plasma analytes that associated with HIV-COPD compared with participants with HIV without COPD after controlling for multiple confounders such as smoking status, race and sex.

We performed LC-MS/MS to identify metabolites of interest and found that 17 of these metabolites were lipids. Among these lipids were diacylglycerol and several sphingolipids. Sphingolipids serve as a structural component of the plasma membrane lipid bilayer and also participate in cell recognition and signalling. In individuals with COPD, increased concentrations of sphingolipids have been measured in sputum. Telenga *et al*^26^ identified 168 sphingolipids and 36 phosphatidylethanolamine lipids in the sputum that were significantly higher in smokers with COPD compared with smokers without COPD. These lipids correlated with reduced lung function, sputum inflammation and smoking history. In our study, we found a complex expression of sphingolipids with both elevated and decreased plasma concentrations of various sphingolipids. This complex plasma expression of sphingolipids was observed by Bowler *et al*, where they used both targeted and untargeted metabolomic platforms that identified 23 sphingolipids common to both platforms in COPD. Some of these lipids correlated to COPD phenotype with five associating with emphysema and seven associating with COPD exacerbations.^26^ ^27^ In addition, sphingolipid expression was complex depending on lipid type and COPD phenotype. Our data support these findings in individuals with HIV, as we found that sphingolipids concentrations were altered in the setting of HIV-COPD when controlling for smoking status.

Among the lipids, we also identified ceramide. Although ceramide concentrations were higher in HIV-COPD, in this relatively small exploratory study, the difference did not quite meet statistical significance (q-value=0.11). Ceramide consists of a sphingolipid backbone (sphingosine) plus a fatty acid. It has been implicated in the pathogenesis of COPD. Petrache *et al*^28^ found that ceramide acts as a second messenger and a crucial mediator of apoptosis and thus alveolar destruction in a murine model of emphysema. In human studies, elevated concentrations of ceramide have been found in sputum and lung tissue.^26^ ^28^ ^29^ Bowler *et al*^27^ further characterised plasma lipids in various COPD phenotypes and found that ceramides had a negative association with emphysema as measured by quantitative high-resolution CT (HRCT); however, trihexosylceramides had a positive association with frequent exacerbators. We also found an elevation of diacylglycerol, a protein kinase C activator that cooperates with ceramide in inducing apoptosis,^30^ but we did not have additional studies, such as HRCT, for further phenotyping.

Lipid dysregulation is a known complication of AIDS and is a well-known side effect of protease inhibitors. Chetwynd *et al*^31^ found that ART resulted in reductions in certain urinary metabolites, including sphingamines. Our study is significant as all of the START participants were ART-naïve and prior to the onset of AIDS. Therefore, the differences in lipid profiles between those with and without COPD cannot be attributed to ART. In addition, lipid abnormalities have been linked to markers of inflammation in HIV compared with healthy controls; however, all of the participants were on HAART and it is unclear whether the lipid abnormalities are due to HIV infection versus ART.^32^ Pertinent to our study, Scarpelini *et al*^33^ identified metabolites that associated with rapid progression of HIV infection and these included sphingomyelin metabolism. It is possible that lipid dysregulation in HIV infection is a marker of worsened disease, including the development of COPD.

In addition to lipids, we identified an analyte consistent with Trp that trended towards an association with HIV-COPD in our metabolite profiling. Trp and its main catabolic enzyme, IDO, have been associated with both COPD and HIV infection.^18^ ^20^ Therefore, we used targeted SRM to measure Trp and its major metabolite, Kyn, to determine if the Trp catabolism is a biomarker for HIV-COPD. Consistent with previous reports, we found decreased Trp and increased Kyn concentrations in individuals with HIV. As a reflection of IDO activity, the Kyn/Trp ratio was significantly elevated in the setting of HIV, but not in COPD participants. Although not statistically significant, we did find that Kyn concentrations were higher than one would predict in those with both COPD and HIV (data not shown). One possibility is that the effects of HIV on IDO activation are much larger than those of COPD, therefore requiring a larger cohort to determine if IDO activation plays a role in HIV-COPD.

There are several limitations to this study. While HIV-positive controls were matched on age, sex, race, region and smoking status to our cases, our non-HIV controls were difficult to match on age and race as our HIV-COPD cases were much younger and over half were black. In this study, we found that metabolites associated with HIV-COPD were not associated with COPD in HIV-negative controls. This may be due to confounders, such as age and race; however, COPD in HIV-negative individuals is most likely a very different phenotype and disease. Therefore, diagnosis and treatment may need to be very different for HIV-COPD and these metabolites have the potential to identify those with HIV who are at risk of developing COPD. This study was relatively small and may have been underpowered to determine all significant biomarkers and metabolomics pathways of HIV-COPD. Future studies would benefit from larger sample sizes and longitudinal study designs such as that recently completed and now possible in the START Pulmonary Substudy.

## Conclusion

HIV-COPD has a unique plasma metabolomic profile that includes sphingolipids and fatty acids. Additional studies are needed to determine how such metabolic pathways contribute to HIV-COPD and whether therapeutic interventions to alter sphingolipid expression can reduce the risk of COPD in PLWH.
