# Supplementary material for: Plasma sphingolipids in HIV-associated chronic obstructive pulmonary disease
Source: BMJ Open Respir Res. 2017 Apr 3;4(1):e000180. doi: 10.1136/bmjresp-2017-000180 (PMC5387954; doi:10.1136/bmjresp-2017-000180)
Supplement: supplementary table 1 [file bmjresp-2017-000180supp_table1.pdf]

Table 1S. Transitions for tryptophan and kynurenine.

Metabolite	Q1 M/Z	Q2 M/Z
Tryptophan	204.892	188
Tryptophan	204.892	169.9
Tryptophan	204.892	158.96
Tryptophan	204.892	117.908
Kynurenine	208.92	191.904
Kynurenine	208.92	145.943
Kynurenine	208.92	135.957
Kynurenine	208.92	94.049
Tryptophan 13C11	216	199
Tryptophan 13C11	216	169
Tryptophan 13C11	216	154
Tryptophan 13C11	216	140.9
Kynurenine D6	215	198
Kynurenine D6	215	150.9
Kynurenine D6	215	142
Kynurenine D6	215	98.2
