# Supplementary material for: Plasma sphingolipids in HIV-associated chronic obstructive pulmonary disease
Source: BMJ Open Respir Res. 2017 Apr 3;4(1):e000180. doi: 10.1136/bmjresp-2017-000180 (PMC5387954; doi:10.1136/bmjresp-2017-000180)
Supplement: supplementary table 2 [file bmjresp-2017-000180supp_table2.pdf]

Table 2S. MS/MS fragmentation data

SPECTRUM - MS
Wendt_Inclu_list_redo_20160328_31.raw
FTMS + p ESI d Full ms2 245.23@hcd37.50 [50.00-270.00]
Scan #: 8905
RT: 18.58

m/z	Intensity	Relative
55.05505	471434.8	18.79
57.07071	458488.4	18.27
67.05497	1544264	61.54
69.0706	779517.4	31.07
79.05485	1222039.6	48.7
81.07049	1976794.3	78.78
83.08608	351587.6	14.01
91.05473	1924314.6	76.69
93.07038	1882180.9	75.01
95.08599	2226375.3	88.73
105.07024	1675198.5	66.76
107.08586	1395408.1	55.61
109.1015	874657.4	34.86
119.08576	1727771.8	68.86
121.10138	1617620.5	64.47
123.11704	324337.7	12.93
133.10126	2509214.8	100
135.11688	774607.5	30.87
137.13245	201871.9	8.05
147.11682	1891404.3	75.38
149.13251	745051.9	29.69
151.1479	138141.4	5.51
161.13251	1909263.6	76.09
163.14812	916929.5	36.54
175.14815	1212218.6	48.31
177.16374	251280.5	10.01
189.1637	528438.4	21.06
203.17915	224742.4	8.96
245.22629	2426380.8	96.7
246.22997	291003.7	11.6

SPECTRUM - MS
Wendt_Inclu_list_redo_20160328_31.raw
FTMS + p ESI d Full ms2 262.25@hcd37.50 [50.00-275.00]
Scan #: 8456
RT: 17.25

m/z	Intensity	Relative
55.05506	350376.8	14.43
57.07074	154996.2	9.37
67.05497	1140686	47
69.0706	349273.9	13
79.05483	1615484.3	78
81.0705	1384085.3	57.85
83.08616	222712.9	10.31
91.05475	678658.5	29.1
93.07037	2328349.3	89
95.0856	1239898.4	50
105.07021	544212.8	20.23
107.08588	1611189.5	100
109.10152	508754.1	22.98
117.07021	199925.4	7.36
119.08572	506725.4	23.65
121.10137	1397288	66.66
123.11692	168782.3	7.25
131.0856	538077.3	25.11
133.10126	436935.7	20.23
135.11687	1152479.8	51.68
145.10112	461877.6	23.65
147.11673	355800.4	18.78
149.13254	755761.2	37.25
159.11685	351236	8.05
161.13246	396522.4	16.56
163.14812	467648.5	21.12
175.14815	322532.4	13.86
189.16101	194315.9	7.52
245.22543	599539.1	25.25
262.25198	915995.3	10.02

SPECTRUM - MS
Wendt_Inclu_list_redo_20160328_31.raw
FTMS + p ESI d Full ms2 261.22@hcd37.50 [50.00-285.00]
Scan #: 8321
RT: 17.37

m/z	Intensity	Relative
55.05506	350376.8	15.05
57.07074	154996.2	6.66
67.05497	1140686	48.99
69.07059	349273.9	15
79.05485	1615484.3	69.38
81.07048	1384085.3	59.44
83.08616	222712.9	9.57
91.05473	678658.5	29.15
93.07037	2328349.3	100
95.08598	1239898.4	53.25
105.07021	544212.8	23.37
107.08588	1611189.5	69.2
109.10152	508754.1	21.85
117.07021	199925.4	8.59
119.08572	506725.4	21.76
121.10137	1397288	60.01
123.11692	168782.3	7.25
131.0856	538077.3	23.11
133.10126	436935.7	18.77
135.11687	1152479.8	49.5
145.10112	461877.6	19.84
147.11673	355800.4	15.28
149.13254	755761.2	32.46
159.11685	351236	15.09
161.13246	396522.4	17.03
163.14812	467648.5	20.08
173.13249	322532.4	13.85
187.14799	194315.9	8.35
243.21068	599539.1	25.75
261.2211	915995.3	39.34

SPECTRUM - MS
Wendt_Inclu_list_redo_20160328_31.raw
FTMS + p ESI d Full ms2 263.24@hcd37.50 [50.00-285.00]
Scan #: 8925
RT: 18.62

m/z	Intensity	Relative
55.05508	2959040	11.47
57.07071	3014287.5	11.69
67.05499	21366134	82.85
69.07064	3495329	13.55
71.0864	1446568.4	5.61
79.05491	4915134	19.06
81.07051	25789230	100
83.08609	3032472.8	11.76
91.05467	3185067.8	12.35
93.07042	5631352	21.84
95.08604	23359914	90.58
97.06515	1287868.5	4.99
97.10139	1358277.3	5.27
99.0809	1439258.3	5.58
105.07028	2556833.8	9.91
107.08591	3622538	14.05
109.10152	12259571	47.54
119.08588	2761468.5	10.71
121.1014	3439529.3	13.34
123.11703	4521524.5	17.53
133.10121	3376571.5	13.09
135.11693	2432434	9.43
137.13225	2073119.8	8.04
147.11687	2554438.3	9.91
149.13251	1541391.6	5.98
151.14825	1410562.6	5.47
161.1326	1899247.4	7.36
175.14832	1625126.5	6.3
245.22658	2705231.3	10.49
263.23712	4750515	18.42

SPECTRUM - MS
Wendt_Inclu_list_redo_20160328_31.raw
FTMS + p ESI d Full ms2 268.26@hcd37.50 [50.00-290.00]
Scan #: 9085
RT: 18.95

m/z	Intensity	Relative
55.05507	3238864.8	59.52
57.07071	3007378.8	55.27
67.05497	1255715.9	23.08
69.07061	5441449.5	100
71.08624	1589165.8	29.2
72.08147	516396.1	9.49
79.05489	731414.6	13.44
81.0705	2169666.3	39.87
83.08612	4473140	82.2
85.1018	665937.7	12.24
86.0606	840645.3	15.45
93.0704	1339954.5	24.62
95.08601	2187015	40.19
97.06529	1149839.3	21.13
97.10165	3659412.3	67.25
100.07616	1391877.9	25.58
107.08592	1087283.1	19.98
109.10152	1002318.6	18.42
111.08078	939313.4	17.26
111.11717	1178814.1	21.66
114.09169	1172487.1	21.55
121.10138	1358247.3	24.96
123.11705	435035.7	7.99
125.09628	574080.8	10.55
128.10709	547783.2	10.07
135.11684	1299076.3	23.87
149.13251	770392	14.16
163.14806	415102.3	7.63
233.22632	610211.1	11.21
268.26303	558135.3	10.26

SPECTRUM - MS
Wendt_Inclu_list_redo_20160328_31.raw
FTMS + p ESI d Full ms2 270.28@hcd37.50 [50.00-295.00]
Scan #: 9652
RT: 20.13

m/z	Intensity	Relative
55.05512	23695.5	2.11
57.03433	5515.9	0.49
57.07073	166840.3	14.86
67.05499	10555.3	0.94
69.07059	37673.3	3.36
71.04987	8622.4	0.77
71.08623	69955.5	6.23
74.06071	70598.5	6.29
81.07035	11452.1	1.02
83.08616	30152.8	2.69
85.10161	24990	2.23
86.06052	15602.4	1.39
88.07623	282442.3	25.16
95.08569	15909.8	1.42
97.06534	8890	0.79
97.10165	22307.3	1.99
100.07623	20044.6	1.79
102.09173	164385	14.65
103.09506	5565.1	0.5
109.10154	10290.6	0.92
114.09167	10870.6	0.97
116.10729	59601.3	5.31
130.1226	20478	1.82
144.13844	11700.4	1.04
186.18599	8306.3	0.74
200.20105	5229	0.47
214.21666	9907.4	0.88
229.23573	10729.2	0.96
270.27902	1122454.9	100
271.28241	148581.7	13.24

SPECTRUM - MS
Wendt_Inclu_list_redo_20160328_31.raw
FTMS + p ESI d Full ms2 280.26@hcd37.50 [50.00-305.00]
Scan #: 8910
RT: 18.59

m/z	Intensity	Relative
55.05507	99185280	54.75
57.07072	45904444	25.34
67.05499	113930672	62.89
69.07062	133580344	73.73
71.08622	17224392	9.51
79.05488	26980374	14.89
81.0705	170047152	93.86
83.08613	98200344	54.2
91.05477	30034526	16.58
93.0704	45853148	25.31
95.08601	181167776	100
97.06524	21168044	11.68
97.10164	64155436	35.41
100.07614	29140808	16.08
105.07024	25809042	14.25
107.08588	35091176	19.37
109.10153	99728984	55.05
111.08076	20361088	11.24
114.0916	24237816	13.38
119.0858	31458360	17.36
121.10144	36962676	20.4
123.11705	44919936	24.79
133.10127	39909800	22.03
135.11693	24342552	13.44
147.11687	30714704	16.95

SPECTRUM - MS
Wendt_Inclu_list_redo_20160328_31.raw
FTMS + p ESI d Full ms2 293.28@hcd37.50 [50.00-320.00]
Scan #: 10494
RT: 21.90

m/z	Intensity	Relative
55.05508	771679.6	61.76
57.03432	48693.8	3.9
57.07071	392911.3	31.45
67.05499	559444.7	44.78
69.07062	1249380.6	100
71.04984	59260.9	4.74
71.08624	184236.9	14.75
79.05483	197219.3	15.79
81.0705	849618.6	68
83.08613	959176.6	76.77
85.06541	62202.4	4.98
85.10181	107829.7	8.63
93.07038	306322.3	24.52
95.08601	912709.1	73.05
97.06528	110026.6	8.81
97.10164	752515.2	60.23
99.08076	67057.9	5.37
107.0859	250977.4	20.09
109.10149	482868.5	38.65
111.08083	128161.9	10.26
111.11718	233675	18.7
121.10141	373955.5	29.93
123.11707	203712.8	16.31
125.09634	52709.5	4.22
125.13284	40082.2	3.21
135.1169	359041.4	28.74
137.13258	66765.5	5.34
149.13255	175239.6	14.03
163.14803	80713	6.46
275.27319	138096.9	11.05

SPECTRUM - MS
Wendt_Inclu_list_redo_20160328_31.raw
FTMS + p ESI d Full ms2 296.26@hcd37.50 [50.00-320.00]
Scan #: 7675
RT: 16.01

m/z	Intensity	Relative
55.01869	398281.5	21.72
55.05507	717448.9	39.13
57.07071	1542926.1	84.16
67.05498	1479151.4	80.68
69.07059	940311	51.29
79.05486	952725.1	51.97
81.0705	1833363.9	100
83.08611	1119458.9	61.06
91.05473	264705.7	14.44
93.07038	1420831.6	77.5
95.086	1717963.3	93.71
97.06525	265688.1	14.49
97.10159	420772.8	22.95
105.07028	157897.7	8.61
107.08588	1261287.6	68.8
109.10152	912937.1	49.8
111.0807	184457.4	10.06
119.08572	193296	10.54
121.10139	1536457.9	83.81
123.117	256531.6	13.99
127.11192	431114.8	23.51
135.11688	1190330.3	64.93
137.13255	179254.7	9.78
149.13251	462033.7	25.2
167.1431	371233.4	20.25
169.12233	589048.8	32.13
233.22633	414900.9	22.63
243.21063	142230.8	7.76
261.22113	176469.7	9.63
279.23206	135914.1	7.41

SPECTRUM - MS
Wendt_Inclu_list_redo_20160328_31.raw
FTMS + p ESI d Full ms2 306.28@hcd37.50 [50.00-330.00]
Scan #: 9161
RT: 19.11

m/z	Intensity	Relative
55.05516	19909.5	14.25
57.07071	11845.6	8.48
67.05501	101240.9	72.44
69.07064	28277.3	20.23
79.05474	21733.3	15.55
81.07052	132501.3	94.8
83.08615	23609.4	16.89
91.05482	12328.9	8.82
93.07034	29694.9	21.25
95.08605	139764.4	100
97.06503	4779.1	3.42
97.10146	4106.2	2.94
100.07624	3649.9	2.61
105.07014	12221.6	8.74
107.08595	31248	22.36
109.10162	73544.2	52.62
114.09191	4189	3
119.08567	13518.7	9.67
121.10154	30816.3	22.05
123.11712	24028.5	17.19
131.08553	11090.5	7.94
133.10085	12254.1	8.77
135.1167	25161	18
137.13245	11217.6	8.03
145.10112	10499.1	7.51
147.11719	4424.9	3.17
149.13245	10254.6	7.34
159.11714	4460.7	3.19
163.14821	4984.8	3.57
173.13194	3719.7	2.66

SPECTRUM - MS
Wendt_Inclu_list_redo_20160328_31.raw
FTMS + p ESI d Full ms2 310.31@hcd37.50 [50.00-335.00]
Scan #: 10489
RT: 21.89

m/z	Intensity	Relative
55.01867	3246148.8	4.39
55.05507	40513496	54.79
57.07071	46038352	62.26
58.06598	1942812.9	2.63
67.05498	18508918	25.03
69.03421	1821665.6	2.46
69.07061	73945576	100
71.04982	2240175.8	3.03
71.08624	26545430	35.9
72.04516	3308114.8	4.47
72.08147	6610563	8.94
79.05486	9796514	13.25
81.07049	32587274	44.07
83.04978	3650415.8	4.94
83.08611	61130828	82.67
85.06532	2299939	3.11
85.10177	11836313	16.01
86.06062	9084905	12.29
86.09702	3180548.5	4.3
93.07037	17767286	24.03
95.08601	32506724	43.96
97.06526	17532448	23.71
97.10162	48729528	65.9
100.07612	19152122	25.9
107.08588	18516182	25.04
109.10152	17474340	23.63
111.08076	14653087	19.82
111.11716	17191728	23.25
114.09162	15231963	20.6
121.1014	19599054	26.5
123.11695	7733754	10.46
125.09624	9572922	12.95
125.13274	3813877.3	5.16
128.10709	6968357.5	9.42
135.11687	20678120	27.96
137.13246	2836658	3.84
139.11183	5335651	7.22
142.1228	3831066.3	5.18

149.13254	12852356	17.38
153.12747	3111889.8	4.21
156.13866	2430454.8	3.29
163.14813	6451422	8.72
167.14333	2174728.5	2.94
177.16377	3241683	4.38
191.1792	1832200.6	2.48
205.19453	1836401	2.48
268.2995	2343487.5	3.17
275.2731	5915956.5	8
293.28433	3799693.5	5.14
310.31003	4940572	6.68

SPECTRUM - MS
Wendt_Inclu_list_redo_20160328_31.raw
FTMS + p ESI d Full ms2 310.31@hcd37.50 [50.00-335.00]
Scan #: 10489
RT: 21.89

m/z	Intensity	Relative
55.01867	3246148.8	4.39
55.05507	40513496	54.79
57.07071	46038352	62.26
58.06598	1942812.9	2.63
67.05498	18508918	25.03
69.03421	1821665.6	2.46
69.07061	73945576	100
71.04982	2240175.8	3.03
71.08624	26545430	35.9
72.04516	3308114.8	4.47
72.08147	6610563	8.94
79.05486	9796514	13.25
81.07049	32587274	44.07
83.04978	3650415.8	4.94
83.08611	61130828	82.67
85.06532	2299939	3.11
85.10177	11836313	16.01
86.06062	9084905	12.29
86.09702	3180548.5	4.3
93.07037	17767286	24.03
95.08601	32506724	43.96
97.06526	17532448	23.71
97.10162	48729528	65.9
100.07612	19152122	25.9
107.08588	18516182	25.04
109.10152	17474340	23.63
111.08076	14653087	19.82
111.11716	17191728	23.25
114.09162	15231963	20.6
121.1014	19599054	26.5
123.11695	7733754	10.46
125.09624	9572922	12.95
125.13274	3813877.3	5.16
128.10709	6968357.5	9.42
135.11687	20678120	27.96
137.13246	2836658	3.84
139.11183	5335651	7.22
142.1228	3831066.3	5.18

149.13254	12852356	17.38
153.12747	3111889.8	4.21
156.13866	2430454.8	3.29
163.14813	6451422	8.72
167.14333	2174728.5	2.94
177.16377	3241683	4.38
191.1792	1832200.6	2.48
205.19453	1836401	2.48
268.2995	2343487.5	3.17
275.2731	5915956.5	8
293.28433	3799693.5	5.14
310.31003	4940572	6.68

SPECTRUM - MS
Wendt_Inclu_list_redo_20160328_31.raw
FTMS + p ESI d Full ms2 338.34@hcd37.50 [50.00-365.00]
Scan #: 11544
RT: 24.11

m/z	Intensity	Relative
53.00301	330071.3	2.63
55.01865	487090.7	3.88
55.05505	6376767	50.79
57.03427	286561.9	2.28
57.07069	8029530	63.95
58.06591	303421	2.42
67.05495	3234627.5	25.76
69.07057	12556307	100
70.0658	283132	2.25
71.04978	405446.9	3.23
71.0862	4980629	39.67
72.04508	497024.8	3.96
72.08144	1043826.6	8.31
79.05483	1603783.6	12.77
81.07046	5542128	44.14
83.04964	655866	5.22
83.08607	10467673	83.37
85.06535	355199.9	2.83
85.10171	2201352.3	17.53
86.06056	1764423.5	14.05
86.09694	512123.4	4.08
91.05464	309770.1	2.47
93.07033	3271885	26.06
95.08597	5933131.5	47.25
97.06522	2783424.8	22.17
97.10158	8150375	64.91
100.07606	3019460	24.05
107.08584	3031829.5	24.15
109.10146	3298934.5	26.27
111.0807	2409507.5	19.19
111.11708	2882569	22.96
114.09157	2632048.3	20.96
121.10133	3431572.5	27.33
123.11696	1470309.3	11.71
125.09623	1561222.6	12.43
125.13262	642877.6	5.12
128.10704	1286367.3	10.24
135.11679	3549902.5	28.27

137.13248	456832.5	3.64
139.11166	1040130.1	8.28
142.12253	676536.9	5.39
149.13245	2129683.3	16.96
153.12732	507996	4.05
156.13823	421720	3.36
163.14807	1066080	8.49
177.16374	563778.9	4.49
191.17931	345935.9	2.76
303.30426	702988.2	5.6
321.31464	522382.8	4.16
338.34134	618028.2	4.92

SPECTRUM - MS
Wendt_Inclu_list_redo_20160328_31.raw
FTMS + p ESI d Full ms2 340.36@hcd37.50 [50.00-365.00]
Scan #: 12579
RT: 26.31

m/z	Intensity	Relative
55.05508	3463.2	13.28
55.07224	1946.8	7.47
57.07066	13584.3	52.09
69.07065	3543.8	13.59
71.08612	5380	20.63
72.08136	2297.9	8.81
74.06071	4746.7	18.2
75.43996	2281.7	8.75
75.98248	2297.8	8.81
78.2285	2594.8	9.95
83.08597	2598.5	9.96
87.50589	2126.6	8.15
88.07626	26077.8	100
95.08584	2877.3	11.03
102.09182	14696	56.35
112.78008	2075.3	7.96
116.10724	3806.7	14.6
143.15794	1967.9	7.55
148.0798	2039.2	7.82
204.05232	2222.8	8.52
235.38141	2340.7	8.98
270.83597	2104.7	8.07

SPECTRUM - MS
Wendt_Inclu_list_redo_20160328_31.raw
FTMS + p ESI d Full ms2 560.33@hcd37.50 [50.00-590.00]
Scan #: 6129
RT: 12.77

m/z	Intensity	Relative
53.89408	2969.9	0.34
56.05028	2807.9	0.32
58.06609	14678.6	1.67
58.27049	2481.3	0.28
60.08158	143525.1	16.32
67.05499	12228.7	1.39
71.07369	40050.6	4.55
79.05513	5634.3	0.64
81.07063	6596.7	0.75
81.26362	3059.5	0.35
82.45902	2859	0.33
86.09699	390280.8	44.37
88.37608	3067.2	0.35
91.05455	21506.5	2.44
93.07024	6572.1	0.75
98.98455	18589.7	2.11
104.10739	756135.4	85.96
104.4232	2963.6	0.34
105.07035	3895.5	0.44
117.0703	12457.5	1.42
125.00002	183399.1	20.85
131.08568	7199.5	0.82
143.52794	4322.7	0.49
145.10049	4105.7	0.47
181.02577	5415.7	0.62
184.07336	879654.4	100
244.58902	4404.1	0.5
268.4704	3213.6	0.37
275.28009	2982.9	0.34
332.67633	2789.7	0.32

SPECTRUM - MS
Wendt_Inclu_list_redo_20160328_31.raw
FTMS + p ESI d Full ms2 613.49@hcd37.50 [50.00-645.00]
Scan #: 7471
RT: 15.58

m/z	Intensity	Relative
51.73846	2472.5	0.17
56.26662	2441.4	0.17
60.90975	2955.7	0.2
76.87393	2858.3	0.2
80.95065	2836.7	0.19
83.54897	2646.8	0.18
97.2464	2624.4	0.18
117.74433	2569	0.18
118.93019	3330.7	0.23
199.9856	3145.7	0.22
243.7502	3125.9	0.21
318.2403	1458926.6	100
415.88837	3289.2	0.23
488.74048	3118.2	0.21
489.52222	3093.3	0.21

SPECTRUM - MS
Wendt_Inclu_list_redo_20160328_33.raw
FTMS + p ESI d Full ms2 854.57@hcd37.50 [59.33-890.00]
Scan #: 12404
RT: 25.94

m/z	Intensity	Relative
60.08154	272955.2	6.27
66.37467	3910.2	0.09
71.07365	153522.7	3.53
77.83513	4474	0.1
86.09695	1509884.1	34.67
91.0545	7646.5	0.18
98.98453	125941.8	2.89
104.10738	113192.5	2.6
124.99995	1029790.1	23.65
146.80617	3930.5	0.09
184.07327	4354984	100
185.07668	7404.6	0.17
297.57477	7428.8	0.17
321.03336	3960.5	0.09
353.10272	4172.4	0.1
377.66391	5616.9	0.13
383.56705	4306.5	0.1
589.11615	4733.2	0.11
636.38177	17411.1	0.4
861.76257	4524.9	0.1
